# LimsPortal and BonsaiLIMS: development of a lab information management system for translational medicine

**DOI:** 10.1186/1751-0473-6-9

**Published:** 2011-05-13

**Authors:** Timothy G Bath, Selcuk Bozdag, Vackar Afzal, Daniel Crowther

**Affiliations:** 1 Translational Medicine Research Collaboration Institute, University of Dundee, Ninewells Hospital, Dundee, DD1 9SY, UK; 2 Translational Medicine Research Collaboration Institute, Pfizer Inc, Ninewells Hospital, Dundee, DD1 9SY, UK

## Abstract

**Background:**

Laboratory Information Management Systems (LIMS) are an increasingly important part of modern laboratory infrastructure. As typically very sophisticated software products, LIMS often require considerable resources to select, deploy and maintain. Larger organisations may have access to specialist IT support to assist with requirements elicitation and software customisation, however smaller groups will often have limited IT support to perform the kind of iterative development that can resolve the difficulties that biologists often have when specifying requirements. Translational medicine aims to accelerate the process of treatment discovery by bringing together multiple disciplines to discover new approaches to treating disease, or novel applications of existing treatments. The diverse set of disciplines and complexity of processing procedures involved, especially with the use of high throughput technologies, bring difficulties in customizing a generic LIMS to provide a single system for managing sample related data within a translational medicine research setting, especially where limited IT support is available.

**Results:**

We have designed and developed a LIMS, BonsaiLIMS, around a very simple data model that can be easily implemented using a variety of technologies, and can be easily extended as specific requirements dictate. A reference implementation using Oracle 11 g database and the Python framework, Django is presented.

**Conclusions:**

By focusing on a minimal feature set and a modular design we have been able to deploy the BonsaiLIMS system very quickly. The benefits to our institute have been the avoidance of the prolonged implementation timescales, budget overruns, scope creep, off-specifications and user fatigue issues that typify many enterprise software implementations. The transition away from using local, uncontrolled records in spreadsheet and paper formats to a centrally held, secured and backed-up database brings the immediate benefits of improved data visibility, audit and overall data quality. The open-source availability of this software allows others to rapidly implement a LIMS which in itself might sufficiently address user requirements. In situations where this software does not meet requirements, it can serve to elicit more accurate specifications from end-users for a more heavyweight LIMS by acting as a demonstrable prototype.

## Background

Within the core laboratory of the Translational Medicine Research Collaboration (TMRC) [1], we routinely profile human samples in order to identify molecular biomarkers. We need to track clinical samples during projects that often use multiple profiling technologies such as Mass Spectrometry based proteomics, ELISA immunoassays and Affymetrix profiling technologies on overlapping patient samples. The tracking of primary clinical samples and derived laboratory samples such as purified mRNA aliquots becomes arduous as the complexity and the sample number increases. Commercial LIMS solutions are available [2] which are not only powerful enough to handle these experimental data sets but are also robust and provide auditing functions to allow experimental labs to meet regulatory requirements. However these vendor solutions also tend to be expensive and require significant technical knowledge to install and run. Different laboratories have very diverse needs from a LIMS ranging from the kind of informal data capture beneficial during academic research to the demonstrable, rigorous adherence to regulatory and governance standards mandated for drug manufacturing and human clinical studies. This means that 'off the shelf' LIMS software with generic functionality require significant customization and tailoring to meet an individual lab's requirements. This customization is difficult, time-consuming and expensive to perform. It is also the case that once a 'generic' LIMS has been tailored to meet a specific lab's working requirements the modifications made, such as modelling novel workflows, tend to be very rigid and do not adapt well to include future lab processes or technologies. In some cases the customisations may not even survive vendor upgrades of the software. In addition, many labs, such as those found in academia or pre-clinical research do not require regulatory compliance but rather need a LIMS which will allow them to manage their samples, clones or strains in an efficient manner in order to facilitate their research. This tracking can be more pressing when samples and results data needs to be shared between labs and research groups in collaborative projects. None of the available LIMS are trivial to deploy, and in larger laboratories LIMS software implementations are as failure-prone as any other large-scale enterprise software implementation project [3].

Translational Medicine aims to improve human health by translating fundamental scientific research into practical applications and thereby bringing new products to market it crosses the traditional clinical/preclinical divide and often involves complex protocol development and modification, multiple platform technologies and generation of diverse raw and processed datasets for analysis. The participants in translational research come from diverse backgrounds have varying levels of computer understanding, and varying degrees of willingness to change their working practises and adopt new software [4]. A bench scientist for example may be accustomed to recording a far greater level of experimental detail than a clinician whose background is emergency patient care, yet both can be involved in the translational research study. As a result of the diversity of disciplines typically found in translational medicine, there is often a proliferation of locally stored data in electronic and paper formats that may not be backed up and may not be stored securely and this poses a risk to any organisation that allows it. This risk is especially pertinent within translational medicine, where management and integration of diverse data can be fundamental to the discovery process. A LIMS therefore is a crucial tool in reducing the risks associated with poor data management in the translational research laboratory.

In this paper we describe BonsaiLIMS, an open source lightweight LIMS system which allows users to manage their studies and sample data though a secure web interface. This has been developed to meet the requirements of our translational research facility and we believe it will have wider utility. We also describe LIMSPortal, a basic portal implementation that includes BonsaiLIMS at its core and other modules to support security and user administration features.

BonsaiLIMS functions as an end solution to provide basic sample tracking capabilities and workflow-specific extensions via the definition of new attribute-value pairs that can be associated with a sample. It is sufficiently simple to replace locally stored spreadsheets and notebook-based records without significant user training or requiring changes to established working practises. The benefits of replacing locally stored lab records with a central database include improved backup and recovery of data and improved reporting and export of data for further analysis.

A useful second function of BonsaiLIMS is that rapid deployment of a very simple/lightweight LIMS can help lay the foundations of a transition to a commercial or more heavy weight LIMS. Getting users out of the habit of storing data locally, defining the data items they wish to capture and discovering features that are liked or disliked all contribute to the understanding of requirements and evaluation criteria for future, more complex systems. The process of implementing a LIMS system will generally require the formalisation of the laboratory processes that the LIMS is to support. This is especially useful in cases where bench scientists may not have prior LIMS experience or be in a position to express comprehensive software requirements [5]. TMRC has a very diverse set of lab-based working processes and modelling all of them in the commercial LIMS system chosen for our lab was not possible in the time available. Therefore an immediate, interim solution was required. BonsaiLIMS uses a modern architecture and flexible object/data model to rapidly deliver a LIMS without the need for significant up-front business analysis, requirements gathering and workflow modelling, while providing users with sufficient LIMS functionality to replace ad-hoc methods of sample tracking and secure the data being generated at the bench.

## Implementation

BonsaiLIMS is implemented as a module that can be embedded into larger portal application. The reason and benefits of this architecture are twofold. Firstly, a module may be adapted and changed independently of the core hosting application. Secondly, it allows for the development of additional functionality in a layered and modular fashion i.e. multiple custom workflows which build upon the base module can be defined and developed in parallel without having any form of mutual dependence. In comparison to other LIMS systems, where implementing custom workflows is often difficult due to the lack of separation or definition of distinct modules, modularity allows for an agile and responsive development process that can more easily adapted to meet the user requirements. The BonaiLIMS module is itself hosted within a portal that allows for a clean separation and integration of the required module functionality. Additional modules hosted within BonsaiLIMS have been developed for authentication, authorisation, security and module deployment.

BonsaiLIMS is implemented using Django, a Python web framework that supports rapid design and development of web based applications [6]. Additional benefits of using Django include support for module based development, increased developer productivity due to built on constructs that provide DRY (Don't Repeat Yourself) functionality and portability across multiple platforms due to the python programming model

**Figure 1:**
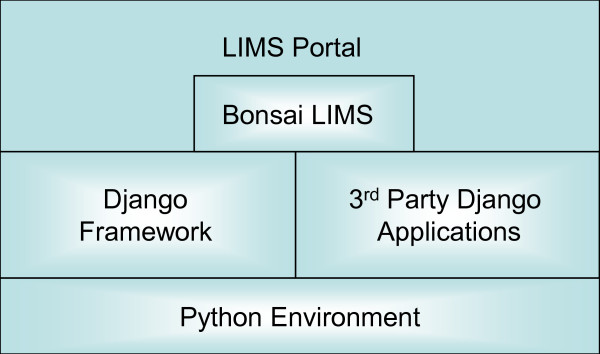
**LimsPortal and BonsaiLIMS **Figure 1 depicts the architecture of a typical Django project. BonsaiLIMS is a loosely coupled Django application which has some dependencies on the main framework and some 3^rd ^party Python libraries. LIMSPortal, on the other hand, is a host "Django project".

**Figure 2:**
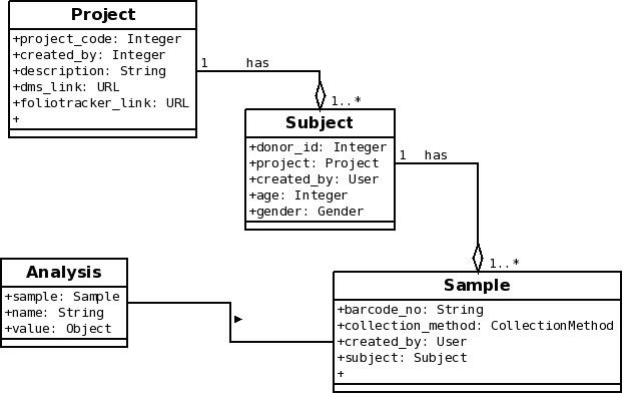
**BonsaiLIMS object model **Project://collection of related samples Subject://donor/source of each sample Sample://material obtained from subject Analysis://operation or process performed on a sample

Figure 1 shows the BonsaiLIMS module within the host LIMSPortal application. LIMSPortal, as part of a Django framework will easily integrate with other 3^rd ^party Django applications and can be deployed wherever there is an available python environment. LIMSportal is a web based solution which is fully HTML and CSS standards compliant and has been tested on Internet Explorer 6/7/8 and Mozilla Firefox 3.x. The basic implementation is built on four object models: Project, Subject, Sample and Analysis. Figure 2 shows the objects and the 1..n relationships between a project and its subjects, a subject and their samples and a sample and its analyses. The data model used by BonsaiLIMS is a relational schema that reflects the object model, with flexible data handling coming from the use of name-value pairs at the analysis level. This removes the need to specify in advance the types of analysis and results that may be entered during a particular workflow.

### Database Integration

**Figure 3:**
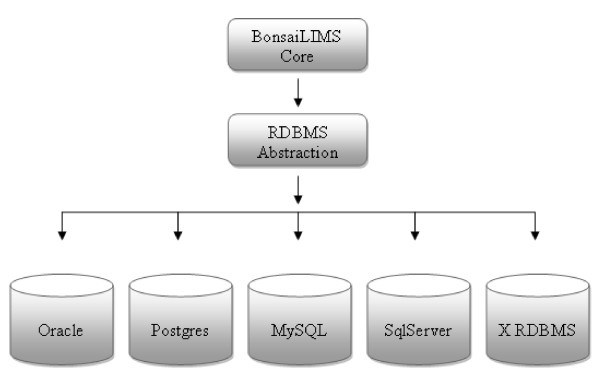
**LimsPortal and BonsaiLIMS **Any relational database can be used for persistence due to abstraction of the data access tier.

The current BonsaiLIMS implementation is backed by an Oracle 11 g instance. However, neither the portal nor BonsaiLIMS utilises any Oracle-specific SQL commands, making them easily portable to other database back ends. Figure 3 illustrates the logical separation of core application functionality and RDBMS data persistence operations, showing how the BonsaiLIMS and LIMSPortal can be deployed with any SQL-compliant database platform. With respect to user auditing, while a popular feature of many commercial, regulated systems we felt that maintaining user audit records, and providing the application functionality to review and manage audit went beyond the lightweight core functionality that we are aiming for with BonsaiLIMS. These are all functions that can be handled very well by the RDBMS and database interaction tier. There is no user export or specific backup functionality provided by the application, as this is readily available directly from the database itself.

### Deploying LIMSPortal

LIMSPortal is deployed on an Apache [7] web server using mod_python module. Static files are deployed on a separate web server to increase the performance. That server runs lighttpd [8] process to serve the files over Internet. Step by step instructions for deployment plus the required python code and Oracle DDL files are in the supplementary material.

### Performance Tuning

LIMSPortal utilises two main approaches for increased performance. The first focuses on reducing the number of requests that must be processed, and the second aims at reducing the size of each request. To reduce the number of requests that must be processed in their entirety, memcached [9] is used to cache the results of HTTP requests. To reduce the size of requests, AJAX is used to enable partial page refreshes.

## Results and Discussion

Due to its simplicity, this architecture and data model enables the rapid deployment of a LIMS without the need for significant preparatory work or ongoing support and maintenance resourcing. Although the reference implementation was developed using Oracle and Django, the concepts can be easily implemented using a variety of software development technologies. The extensibility of the model allows future protocols and data items to be added by end-users with no reprogramming. It provides lab data management and helps integrate platform technologies with very little user training or changes to scientists existing working procedures being required. The implementation presented describes a portal platform and LIMS component that have been extended to provide additional functionality such as LDAP integration for user administration and authentication. Although LDAP was chosen as most appropriate for the translation medicine research collaboration smaller experimental groups who might benefit most from BonsaiLIMS may even prefer the simpler option of database or application authentication.

### Functionality

The system is supporting the diverse workflows used by TMRC Genomics, Immunoassay and Tissue Culture groups. Data is being recorded centrally and ad-hoc methods involving spreadsheets are being phased out. In addition, by capturing lab process data in this structured way, migration to a more heavyweight LIMS is eased from both user and data perspectives.

### Performance

The application is stable, robust and responsive. For enhancing data capture, GUI enhancements would improve usability especially with regard to batch data entry. Typical usage is that bench work is performed and hand written notes are made in a lab notebook, and then important results are input to LIMS. Closer inspection of this process has identified that the data entry screens are not optimal for much more than single data item entry or updates.

### Comparison with similar software

There are many open source [10,11] and commercial [12] LIMS systems available that demand significant investment of time and money in order to obtain the promised return. These tend to be feature-rich, heavyweight systems that offer generic functionality that can be tailored to a specific labs needs, or are focused on a very specific lab function such as Proteomics analysis or Microarray studies [13]. However, no LIMS could be found that combined the portal-based hosting framework and combination of modules to deliver specific functionality that this paper describes.

### Intended use & benefits

LIMSPortal achieves goal of moving bench scientists involved in translational research away from ad-hoc data recording and facilitates central management of lab data along with the benefits of improved sample management and collaboration between lab scientists.

## Conclusions

LIMS are complicated systems and are not trivial to implement, especially in the translational research laboratory. TMRC had pressing requirements for immediate, simple lab data management and sample tracking and the LIMSPortal/BonsaiLIMS model met these requirements, evidenced by its uptake by Immunoassay, Tissue Culture and Genomics groups. The system was operational and capturing data within 3 weeks of conception and has now been back-populated with historical data previously held on spreadsheets. Lab data is now held in uniform, structured electronic format, and more readily available for analysis. While the LIMS is feature poor in comparison to other LIMS systems available, for example it does not track user activity for audit purposes, it is an effective interim solution to TMRC's lab data management and data integration needs. The migration from a lightweight LIMS such as BonsaiLIMS to a more powerful system is made far easier, as stakeholders have a baseline for comparison and have moved away from a culture of local document and data storage. LIMSPortal and BonsaiLIMS are currently deployed at TMRC and are currently managing data for 67 projects and 28,456 samples and aliquots.

## Availablity and Requirements

The BonsaiLIMS instance installed at TMRC contains commercially sensitive information and is not publicly available. However, the Python files, database schema creation scripts, a user guide and instructions on how to deploy can all be found in the additional materials files submitted with this manuscript (Additional files 1, 2, 3, 4, 5) and on the BonsaiLIMS sourceforge page.

• **Project name**: LimsPortal

• **Project home page**: http://bonsailims.sourceforge.net/

• **Operating system(s)**: Platform independent

• **Programming language**: Python/Django

• **Other requirements**: Apache webserver, mod_python, lighttpd

• **License**: GNU LGPL

• **Any restrictions to use by non-academics**: None

## List of Abbreviations used

AJAX: Asynchronous JavaScript and XML; CSV: Comma Separated Value; GUI: Graphical User Interface; LDAP: Lightweight Directory Access Protocol; LIMS: Laboratory Information Management Systems; TMRC: Translational Medicine Research Collaboration.

## Competing interests

SB, VA and TB have no competing interests. DC is an employee of Pfizer Inc.

## Authors' contributions

DC co-ordinated the project, SB chose the technology platform and implemented the design in Django, TB implemented data model in Oracle, VA was responsible for the installation and configuring of the web server components. All authors contributed to the final manuscript.

## Supplementary Material

Additional file 1 **Bonsai Deployment.doc **Instructions how to deploy BonsaiLIMS Click here for file

Additional file 2 **bonsai.zip **Compressed file containing the python source code for BonsaiLIMS Click here for file

Additional file 3 **BonsaiLIMS_DDL_Oracle11g.sql **SQL script to recreate oracle database schema Click here for file

Additional file 4 **site_media.zip **Graphics required for web GUI interface elements Click here for file

Additional file 5 **BonsaiLIMS user guide.doc **A brief description of some of the BonsaiLIMS functionality Click here for file

## References

[B1] Translational Medicine Research Collaboration http://news.bbc.co.uk/1/hi/scotland/tayside_and_central/8010580.stm

[B2] Ekins S Computer applications in pharmaceutical research and development. Wiley 58 60

[B3] McDowall RD Risk management for laboratory automation projects Journal of the Association for Laboratory Automation 2004 9 2 72 86 10.1016/j.jala.2004.01.002

[B4] Plebani Mario The changing scenario in laboratory medicine and the role of laboratory professionals in translational medicine Clinica Chimica Acta 2008 393 1 23 26 10.1016/j.cca.2008.03.013 18423398

[B5] Broad LA Maloney TA Subak EJ Jr Selection of Lims for a Pharmaceutical Research and Development Laboratory--A Case Study Data Handling in Science and Technology 1990 6 315 327

[B6] Reuven Lerner M At the forge: First steps with Django Linux Journal 159 11

[B7] Laurie Ben Laurie Peter Apache: The Definitive Guide 3 O'Reilly & Associates, Inc

[B8] Bogus Andre Lighttpd 2008 Packt Publishing

[B9] Fitzpatrick Brad Distributed caching with memcached Linux Journal 2004 124

[B10] Viksna Juris Celms Edgars Opmanis Martins Podnieks Karlis Rucevskis Peteris Zarins Andris Barrett Amy Sudeshna Guha Neogi Krestyaninova Maria Mark McCarthy I Brazma Alvis Ugis Sarkans PASSIM - an open source software system for managing information in biomedical studies BMC Bioinformatics 2007 8 52 10.1186/1471-2105-8-52 17291344 PMC1803798

[B11] FreeLIMS http://sourceforge.net/projects/freelims/by fina

[B12] Piggee Christine LIMS and the art of MS proteomics Anal. Chem 2008 80 13 4801 4806 10.1021/ac0861329 18609747

[B13] Maurer Michael Molidor Robert Sturn Alexander Hartler Juergen Hackl Hubert Stocker Gernot Prokesch Andreas Scheideler Marcel Trajanoski Zlatko MARS: Microarray analysis, retrieval, and storage system BMC Bioinformatics 2005 6 101 10.1186/1471-2105-6-101 15836795 PMC1090551

