# Supplementary material for: LimsPortal and BonsaiLIMS: development of a lab information management system for translational medicine
Source: Source Code Biol Med. 2011 May 13;6:9. doi: 10.1186/1751-0473-6-9 (PMC3113716; doi:10.1186/1751-0473-6-9)
Supplement: Additional file 1 — Bonsai Deployment.doc Instructions how to deploy BonsaiLIMS [file 1751-0473-6-9-S1.DOC]

# Bonsai Deployment

| Contents [[hide](javascript:toggleToc())]   - [1](http://wiki.tmrc.co.uk/tmrcwiki/index.php/Bonsai_Deployment#Deploying_BonsaiLIMS) Deploying BonsaiLIMS   - [1.1](http://wiki.tmrc.co.uk/tmrcwiki/index.php/Bonsai_Deployment#Introduction) Introduction   - [1.2](http://wiki.tmrc.co.uk/tmrcwiki/index.php/Bonsai_Deployment#Environment) Environment   - [1.3](http://wiki.tmrc.co.uk/tmrcwiki/index.php/Bonsai_Deployment#Installation_Steps) Installation Steps   - [1.4](http://wiki.tmrc.co.uk/tmrcwiki/index.php/Bonsai_Deployment#Installing_the_Sources) Installing the Sources   - [1.5](http://wiki.tmrc.co.uk/tmrcwiki/index.php/Bonsai_Deployment#Configuring_Apache) Configuring Apache   - [1.6](http://wiki.tmrc.co.uk/tmrcwiki/index.php/Bonsai_Deployment#Post_Installation) Post Installation |
| --- |

## 1 Deploying BonsaiLIMS

### 1.1 Introduction

This document intends to give details about deploying BonsaiLIMS. BonsaiLIMS is a web application powered by Django([http://www.djangoproject.com](http://www.djangoproject.com/)) development environment. Django is a modern, easy-to-use web programming framework written in Python programming language.

Steps given below are tested on tmrc-django-dev server.

### 1.2 Environment

- Basic Installation
  - Centos 5
  - Apache 2.2
  - Python 2.4.3
- Sources
  - bonsai.zip
  - site_media.zip
- Dependencies
  - Django 1.0.2
  - mod_python
  - Oracle Instant Client 11.1 Basic
  - cx_Oracle
  - python-ldap
  - setuptools
  - simplejson

### 1.3 Installation Steps

**(!) Warning** All commands require "sudo" in front.

1. It is assumed that Centos5+Apache+Python are already installed.
2. Download Django 1.0.2 on <http://www.djangoproject.com/download/> and follow the steps there.
3. **mod_python** can be installed using repository.
4. Execute BonsaiLIMS_DDL_Oracle11g.sql as the schema owner of the Oracle database where you are deploying the software.

yum install mod_python

You do not need to reconfigure Apache afterwards.

1. Oracle Instance Client 11.1 Basic is available in RPM format on <http://www.oracle.com/technology/software/tech/oci/instantclient/index.html>. After downloading the RPM, you can install using yum again.

yum install oracle-instantclient11.1-basic-11.1.0.7.0-1.i386.rpm --nogpgcheck

This installation will install libraries under /usr/lib/oracle/11.1/client/lib. I experienced a problem while putting them in the sys.path. For the sake of simplicity(may not be elegant), all files are **copied** to /usr/lib.

1. cx_Oracle is the bridge between Oracle and Python.

An RPM package is available on <http://prdownloads.sourceforge.net/cx-oracle/cx_Oracle-5.0.1-11g-py24-1.i386.rpm?download> . That version is compiled using Python 2.4 for accessing Oracle 11g which is our case.

yum install cx_Oracle-5.0.1-11g-py24-1.i386.rpm --nogpgcheck

This will install all necessary libraries and sources.

**(i) CHECK POINT**

$ python

>> import cx_Oracle

>>

Try python shell to include cx_Oracle module. You should not get any error messages or warnings.

1. python-ldap is a package again available inside standard yum repository.

yum install python-ldap

1. setuptools can be installed from repo.

yum install python-setuptools

1. simplejson can be installed from repo.

yum install python-simplejson

### 1.4 Installing the Sources

1. Unzip bonsai.zip under "/opt". (i.e. directory will be /opt/bonsai)
2. Unzip site_media.zip under your Apache server's root folder (e.g. /var/www/html/site_media)

### 1.5 Configuring Apache

Create the file below and place it under /etc/httpd/conf.d.

bonsai.conf

<Location "/">

SetHandler python-program

PythonHandler django.core.handlers.modpython

SetEnv DJANGO_SETTINGS_MODULE bonsai.settings

PythonOption django.root /bonsai

PythonPath "['/opt', '/opt/bonsai'] + sys.path"

PythonDebug On

</Location>

<Location "/site_media">

SetHandler None

</Location>

### 1.6 Post Installation

Restart the Apache using "/sbin/service httpd restart".
