# Supplementary material for: LimsPortal and BonsaiLIMS: development of a lab information management system for translational medicine
Source: Source Code Biol Med. 2011 May 13;6:9. doi: 10.1186/1751-0473-6-9 (PMC3113716; doi:10.1186/1751-0473-6-9)
Supplement: Additional file 2 — bonsai.zip Compressed file containing the python source code for BonsaiLIMS [file 1751-0473-6-9-S2.zip › bonsai/templates/analysis/add_attr.html]

{%extends 'base.html'%}
{%block extrahead%}

{%endblock%}
{%block contentcolumn%}

Analysis » Add Attribute

{% if form.errors %}
**Solve the errors below**
{% endif %}
Add New Analysis Type


|  |  |
| --- | --- |
|{{form.as\_table}}|  |  |
| --- | --- |
|  |  |

### Help

Sample list is generated only from the projects you subscribed.
*Name* is autocompleted by looking at the *Analysis Type*.

### Where am I?

- Analysis
  - Create Attribute

{%if success %}
**Saved successfully!**
{%endif%}

{%endblock%}
