# Supplementary material for: LimsPortal and BonsaiLIMS: development of a lab information management system for translational medicine
Source: Source Code Biol Med. 2011 May 13;6:9. doi: 10.1186/1751-0473-6-9 (PMC3113716; doi:10.1186/1751-0473-6-9)
Supplement: Additional file 2 — bonsai.zip Compressed file containing the python source code for BonsaiLIMS [file 1751-0473-6-9-S2.zip › bonsai/templates/analysis/analysis_add.html]

 

|  |  |
| --- | --- |
| Analysis Type: | {{form.analysis\_type}} | Or Add a new type |
| Analysis Attribute: | {{form.name}} |
| Attribute Value: | {{form.value}} |
| Which aliquot: | |  | | --- | |{%for k,v in aliquot\_dict.items %} {%if not v%} {%else%} {%endif%} |{%endfor%} |{%for k,v in aliquot\_dict.items %} {%if not v%} {%else%} {%endif%} |{%endfor%} |{%for k,v in aliquot\_dict.items %} {%if not v%} {{k}} {%else%} **{{k}}** {%endif%} |{%endfor%} |
|  |  |
