# Supplementary material for: LimsPortal and BonsaiLIMS: development of a lab information management system for translational medicine
Source: Source Code Biol Med. 2011 May 13;6:9. doi: 10.1186/1751-0473-6-9 (PMC3113716; doi:10.1186/1751-0473-6-9)
Supplement: Additional file 2 — bonsai.zip Compressed file containing the python source code for BonsaiLIMS [file 1751-0473-6-9-S2.zip › bonsai/templates/analysis/delete.html]

{%extends 'base.html'%}
{%block contentcolumn%}

Analysis » Delete

**Are you sure to continue to delete this analysis?**

### Help

All analysis data will be removed permanently. Please be aware of what you are doing.

### Where am I?

- Analysis
  - Delete

{%endblock%}
