# Supplementary material for: LimsPortal and BonsaiLIMS: development of a lab information management system for translational medicine
Source: Source Code Biol Med. 2011 May 13;6:9. doi: 10.1186/1751-0473-6-9 (PMC3113716; doi:10.1186/1751-0473-6-9)
Supplement: Additional file 2 — bonsai.zip Compressed file containing the python source code for BonsaiLIMS [file 1751-0473-6-9-S2.zip › bonsai/templates/analysis/show_by_sample.html]

{%extends 'base.html'%}
{%load core\_extras%}
{%block title%}Show analysis for {{sample}}{%endblock%}
{%block contentcolumn%}
{%ifequal analyses.count 0%}
No analysis found. Add a new analysis from "Create » New Analysis".
{%else%}
Filter when value is empty?

| Aliquot Id | Analysis Type | Attribue | Value | Last Updated By | Date Last Updated | Time Last Updated |
| --- | --- | --- | --- | --- | --- | --- |
{%for analysis in analyses %}| {{analysis.aliquot\_id}} | {{analysis.analysis\_type}} | {{analysis.name}} | {{analysis.value}} | {{analysis.last\_updated\_by.first\_name}} | {{analysis.date\_time\_last\_updated|date}} | {{analysis.date\_time\_last\_updated|time}} |
{%endfor%}

{%if page.has\_previous %}
<< Prev
{%endif%}
{{page}}
{%if page.has\_next %}
Next >>
{%endif%}

{%endifequal%}
{%endblock%}
