# Supplementary material for: LimsPortal and BonsaiLIMS: development of a lab information management system for translational medicine
Source: Source Code Biol Med. 2011 May 13;6:9. doi: 10.1186/1751-0473-6-9 (PMC3113716; doi:10.1186/1751-0473-6-9)
Supplement: Additional file 2 — bonsai.zip Compressed file containing the python source code for BonsaiLIMS [file 1751-0473-6-9-S2.zip › bonsai/templates/base.html]

Bonsai LIMS / {%block title%} {%endblock%}


{%block extrahead%}
{%endblock%}


BonsaiLIMSbeta

{%if request.user.is\_authenticated %}
**Hello {{request.user.first\_name}} | Settings | Logout |**
{%endif%}
Help

{%if request.user.is\_authenticated %}

- Bookmarks
- Create
- Samples Worklist
- Subjects Worklist
- My Projects
- All Projects

- New Analysis
- New Sample
- New Subject

- Subjects
- Samples

Sample:

{%endif%}

{%block contentcolumn%}
{%endblock%}

{% if messages %}

{% for message in messages %}- **{{ message }}**
{% endfor %}
{% endif %}
©2009 TMRC | About | Help
