# Supplementary material for: LimsPortal and BonsaiLIMS: development of a lab information management system for translational medicine
Source: Source Code Biol Med. 2011 May 13;6:9. doi: 10.1186/1751-0473-6-9 (PMC3113716; doi:10.1186/1751-0473-6-9)
Supplement: Additional file 2 — bonsai.zip Compressed file containing the python source code for BonsaiLIMS [file 1751-0473-6-9-S2.zip › bonsai/templates/index.html]

{% extends 'base.html' %}
{%block title%}Home{%endblock%}
{%block content%}{%endblock%}
