# Supplementary material for: LimsPortal and BonsaiLIMS: development of a lab information management system for translational medicine
Source: Source Code Biol Med. 2011 May 13;6:9. doi: 10.1186/1751-0473-6-9 (PMC3113716; doi:10.1186/1751-0473-6-9)
Supplement: Additional file 2 — bonsai.zip Compressed file containing the python source code for BonsaiLIMS [file 1751-0473-6-9-S2.zip › bonsai/templates/login.html]

{%extends 'base.html'%}
{%block title%}Login{%endblock%}
{%block contentcolumn%}

|  |  |
| --- | --- |
| Username: |  |
| Password: |  |
|  | | Forgot your username/password? |

## Welcome to Bonsai LIMS

Bonsai LIMS is the study, sample and results management system designed for TMRC.

## Why Bonsai LIMS?

> Bonsai is the art of aesthetic miniaturisation of trees.   
> *Wikipedia*

Bonsai LIMS is an aesthetic miniaturisation of ATLAS/SQL\*LIMS, specifically designed for TMRC.

## See what it's all about!

To get started, please login using the box on the top.

{%endblock%}
