# Supplementary material for: LimsPortal and BonsaiLIMS: development of a lab information management system for translational medicine
Source: Source Code Biol Med. 2011 May 13;6:9. doi: 10.1186/1751-0473-6-9 (PMC3113716; doi:10.1186/1751-0473-6-9)
Supplement: Additional file 2 — bonsai.zip Compressed file containing the python source code for BonsaiLIMS [file 1751-0473-6-9-S2.zip › bonsai/templates/projects/add.html]

{%extends 'project\_perspective.html'%}
{% block centerpane %}

{% if error %}

Invalid form

{% else %}

Project saved successfully

{% endif %}

{{form.as\_table}}

{% endblock %}
