# Supplementary material for: LimsPortal and BonsaiLIMS: development of a lab information management system for translational medicine
Source: Source Code Biol Med. 2011 May 13;6:9. doi: 10.1186/1751-0473-6-9 (PMC3113716; doi:10.1186/1751-0473-6-9)
Supplement: Additional file 2 — bonsai.zip Compressed file containing the python source code for BonsaiLIMS [file 1751-0473-6-9-S2.zip › bonsai/templates/projects/list_subscribed.html]

{%extends 'base.html'%}
{%load core\_extras%}
{%block title%}All Projects{%endblock%}
{%block contentcolumn%}

| Project | Description | Sample No | Subjects | Samples |
| --- | --- | --- | --- | --- |
{% for subscription in subscriptions %}| {{subscription}} | {{subscription.description|truncatewords:5}} | {{subscription|sample\_count}} | Show({{subscription.subjects.count}}) | Show({{subscription|sample\_count}}) |
{% endfor %}

{{subscriptions.count}} projects are subscribed.

Click on bells to get notifications about the project.
Learn Why?

{%endblock%}
