# Supplementary material for: LimsPortal and BonsaiLIMS: development of a lab information management system for translational medicine
Source: Source Code Biol Med. 2011 May 13;6:9. doi: 10.1186/1751-0473-6-9 (PMC3113716; doi:10.1186/1751-0473-6-9)
Supplement: Additional file 2 — bonsai.zip Compressed file containing the python source code for BonsaiLIMS [file 1751-0473-6-9-S2.zip › bonsai/templates/projects/samples.html]

{%extends 'subject\_perspective.html'%}
{% block centerpane %}

## Listing all samples of {{project.project\_code}}

{%if error %}

No sample found!

{%else%}

| Edit | Subject | Barcode No | Aliquot No | Collection Method | Date/Time Collected | Date/Time Destroyed | Date/Time Frozen | External Sample Id | Material | Freezer Location | Freeze Method | Storage Method | DMS |
| --- | --- | --- | --- | --- | --- | --- | --- | --- | --- | --- | --- | --- | --- |
{%for sample in page\_items.object\_list%}|  | {{sample.subject}} | {{sample.barcode\_no}} | {{sample.aliquot\_no}} | {{sample.collection\_method}} | {{sample.date\_time\_collected}} | {{sample.date\_time\_destroyed}} | {{sample.date\_time\_frozen}} | {{sample.external\_sample\_id}} | {{sample.material}} | {{sample.freezer\_location}} | {{sample.freeze\_method}} | {{sample.storage\_method}} | {{sample.dms}} |
{%endfor%}

{%if page\_items.has\_previous %}
Previous
{% endif %}
{{page\_items}}
{%if page\_items.has\_other\_pages %}
Next
{% endif %}

{% endif %}
{% endblock %}
