# Supplementary material for: LimsPortal and BonsaiLIMS: development of a lab information management system for translational medicine
Source: Source Code Biol Med. 2011 May 13;6:9. doi: 10.1186/1751-0473-6-9 (PMC3113716; doi:10.1186/1751-0473-6-9)
Supplement: Additional file 2 — bonsai.zip Compressed file containing the python source code for BonsaiLIMS [file 1751-0473-6-9-S2.zip › bonsai/templates/projects/show.html]

{%extends 'subject\_perspective.html'%}
{% block centerpane %}

## {{project.project\_code}}

### *Description* {{project.description}} *Related Links* - DMS1{{project.dms\_link}} - FolioTracker2{{project.foliotracker\_link}} *Log* This information is last updated on {{project.date\_time\_last\_updated|date}} at {{project.date\_time\_last\_updated|time}} by {{project.last\_updated\_by}}. {% endblock %}
