# Supplementary material for: LimsPortal and BonsaiLIMS: development of a lab information management system for translational medicine
Source: Source Code Biol Med. 2011 May 13;6:9. doi: 10.1186/1751-0473-6-9 (PMC3113716; doi:10.1186/1751-0473-6-9)
Supplement: Additional file 2 — bonsai.zip Compressed file containing the python source code for BonsaiLIMS [file 1751-0473-6-9-S2.zip › bonsai/templates/samples/add.html]

{% extends 'base.html'%}
{% block contentcolumn %}

Samples » Add Sample

{% if form.errors %}

Please correct the error{{ form.errors|pluralize }} below.

{% endif %}

{{form.as\_table}}|  |  |
| --- | --- |
|  |  |

### Info

You are only allowed to add samples under subjects of the projects you subscribed before. The reason you cannot
list all the projects under the projects combobox is to make you focus on projects only you want to
deal with. You can always subscribe to other projects as well as you can unsubscribe from the ones you have
already done with. That will increase your performance and reduce the risk of making mistakes.
Do you think it is not a good idea or have a better idea? Then send us your comments.

### Where am I?

- Samples
  - Create New

{% endblock %}
