# Supplementary material for: LimsPortal and BonsaiLIMS: development of a lab information management system for translational medicine
Source: Source Code Biol Med. 2011 May 13;6:9. doi: 10.1186/1751-0473-6-9 (PMC3113716; doi:10.1186/1751-0473-6-9)
Supplement: Additional file 2 — bonsai.zip Compressed file containing the python source code for BonsaiLIMS [file 1751-0473-6-9-S2.zip › bonsai/templates/samples/show.html]

|  |  |
| --- | --- |
| Subject: | {{sample.subject}} |
| Barcode No: | {{sample.barcode\_no}} |
| Aliquot No: | {{sample.aliquot\_no}} |
| Collection Method: | {% if sample.collection\_method %} {{sample.collection\_method}} {% else %} N/A {% endif %} |
| Date/Time Collected: | {% if sample.date\_time\_collected %} {{sample.date\_time\_collected|date}} at {{sample.date\_time\_collected|time}} {% else %} N/A {% endif %} |
| Date/Time Destroyed: | {% if sample.date\_time\_destroyed %} {{sample.date\_time\_destroyed|date}} at {{sample.date\_time\_destroyed|time}} {% else %} N/A {% endif %} |
| DMS Sample Info Sheet On: | {% if sample.dms\_sample\_info\_sheet\_link %} {{sample.dms\_sample\_info\_sheet\_link}} {% else %} N/A {% endif %} |
| External Sample Id: | {% if sample.external\_sample\_id %} {{sample.external\_sample\_id}} {% else %} N/A {% endif %} |
| Material: | {% if sample.material %} {{sample.material}} {% else %} N/A {% endif %} |
| Storage Method: | {% if sample.storage\_method %} {{sample.storage\_method}} {% else %} N/A {% endif %} |
| Freezer Location: | {% if sample.freezer\_location %} {{sample.freezer\_location}} {% else %} N/A {% endif %} |
| Freezer Method: | {% if sample.freeze\_method %} {{sample.freeze\_method}} {% else %} N/A {% endif %} |

Last updated on **{{sample.date\_time\_last\_updated|date}}** at **{{sample.date\_time\_last\_updated|time}}** by **{{sample.last\_updated\_by}}**.
