# Supplementary material for: LimsPortal and BonsaiLIMS: development of a lab information management system for translational medicine
Source: Source Code Biol Med. 2011 May 13;6:9. doi: 10.1186/1751-0473-6-9 (PMC3113716; doi:10.1186/1751-0473-6-9)
Supplement: Additional file 2 — bonsai.zip Compressed file containing the python source code for BonsaiLIMS [file 1751-0473-6-9-S2.zip › bonsai/templates/samples/show_by_subject.html]

{%extends 'base.html'%}
{%load core\_extras%}
{%block title%}Samples by subject: {{subject}}{%endblock%}
{%block extrahead%}
{% endblock %}

Samples » List by Subject » {{subject}}

{%block contentcolumn%}

| Barcode No | Subject | Collection Method | Freezer Location | Material | Aliquot No | More |
| --- | --- | --- | --- | --- | --- | --- |
{%for sample in samples %}| {%if sample\_bookmarks|is\_in\_sample\_bookmarks:sample %}  {%else%}  {%endif%}    {{sample.barcode\_no}} | {{sample.subject}} | {{sample.collection\_method}} | {{sample.freezer\_location}} | {{sample.material}} | {{sample.aliquot\_no}} | Show |
{%endfor%}

{%if page.has\_previous %}
<< Prev
{%endif%}
{{page}}
{%if page.has\_next %}
Next >>
{%endif%}

Goto »
{% for i in paginator.page\_range %}
{%ifnotequal i page.number%}
{{i}}
{%else%}
{{i}}
{%endifnotequal%}
{% endfor %}

{%endblock%}
