# Supplementary material for: LimsPortal and BonsaiLIMS: development of a lab information management system for translational medicine
Source: Source Code Biol Med. 2011 May 13;6:9. doi: 10.1186/1751-0473-6-9 (PMC3113716; doi:10.1186/1751-0473-6-9)
Supplement: Additional file 2 — bonsai.zip Compressed file containing the python source code for BonsaiLIMS [file 1751-0473-6-9-S2.zip › bonsai/templates/subjects/mysubjects.html]

{%extends 'base.html'%}
{%load core\_extras%}
{%block title%}Subjects you updated{%endblock%}
{%block extrahead%}
{% endblock %}
{%block contentcolumn%}

| Subject *[Total: {{paginator.count}}]* | Age | Gender | Samples |
| --- | --- | --- | --- |
{%for subject in subjects %}| {%if subject\_bookmarks|is\_in\_subject\_bookmarks:subject %}  {%else%}  {%endif%}   {{subject}} | {{subject.age}} | {{subject.gender}} | {{subject.samples.count}} |
{%endfor%}

{%if page.has\_previous %}
<< Prev
{%endif%}
{{page}}
{%if page.has\_next %}
Next >>
{%endif%}

Goto »
{% for i in paginator.page\_range %}
{%ifnotequal i page.number%}
{{i}}
{%else%}
{{i}}
{%endifnotequal%}
{% endfor %}

Click on stars to subscribe to or unsubscribe from projects.
Learn Why?

{%endblock%}
