# Supplementary material for: LimsPortal and BonsaiLIMS: development of a lab information management system for translational medicine
Source: Source Code Biol Med. 2011 May 13;6:9. doi: 10.1186/1751-0473-6-9 (PMC3113716; doi:10.1186/1751-0473-6-9)
Supplement: Additional file 2 — bonsai.zip Compressed file containing the python source code for BonsaiLIMS [file 1751-0473-6-9-S2.zip › bonsai/templates/subjects/show.html]

{% extends 'sample\_perspective.html'%}
{% block centerpane %}

## Subject: {{subject.donor\_id}}

{% ifequal subject.gender "M" %}
{% else %}
{% ifequal subject.gender "F" %}
{% else %}
{% endifequal %}
{%endifequal%}

### *Information*

> Age: {%if subject.age %}
> {{subject.age}}
> {%else%}
> N/A
> {%endif%}
>   
> Gender:
> {%ifequal subject.gender "M"%}
> Male
> {%else%}{%ifequal subject.gender "F"%}
> Female
> {%else%}
> N/A
> {%endifequal%}
> {%endifequal%}
>   
> A subject of project {{subject.project}}.

### *Log*

This information is last updated on
{{subject.date\_time\_last\_updated|date}} at {{subject.date\_time\_last\_updated|time}} by
{{subject.last\_updated\_by}}.

{% endblock %}
