# Supplementary material for: LimsPortal and BonsaiLIMS: development of a lab information management system for translational medicine
Source: Source Code Biol Med. 2011 May 13;6:9. doi: 10.1186/1751-0473-6-9 (PMC3113716; doi:10.1186/1751-0473-6-9)
Supplement: Additional file 2 — bonsai.zip Compressed file containing the python source code for BonsaiLIMS [file 1751-0473-6-9-S2.zip › bonsai/templates/user_profile.html]

{%extends 'base.html'%}
{%block title%}Settings for {{request.user}} {%endblock%}
{%block contentcolumn%}

{{request.user}} » Settings

{% if form.errors %} **Solve the errors below** {% endif %}
{{form.as\_table}}

|  |  |
| --- | --- |
|  |  |

### Help

You can change some defaults using the form on the left.

### Where am I?

- {{request.user}}
  - Settings

{%endblock%}
