# Supplementary figures and images for: LimsPortal and BonsaiLIMS: development of a lab information management system for translational medicine
Source: Source Code Biol Med. 2011 May 13;6:9. doi: 10.1186/1751-0473-6-9 (PMC3113716; doi:10.1186/1751-0473-6-9)

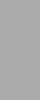

Supplement: Additional file 4 — site_media.zip Graphics required for web GUI interface elements [file 1751-0473-6-9-S4.ZIP › site_media/css/smoothness/images/ui-bg_flat_0_aaaaaa_40x100.png]

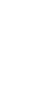

Supplement: Additional file 4 — site_media.zip Graphics required for web GUI interface elements [file 1751-0473-6-9-S4.ZIP › site_media/css/smoothness/images/ui-bg_flat_75_ffffff_40x100.png]

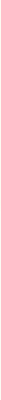

Supplement: Additional file 4 — site_media.zip Graphics required for web GUI interface elements [file 1751-0473-6-9-S4.ZIP › site_media/css/smoothness/images/ui-bg_glass_55_fbf9ee_1x400.png]

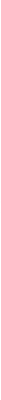

Supplement: Additional file 4 — site_media.zip Graphics required for web GUI interface elements [file 1751-0473-6-9-S4.ZIP › site_media/css/smoothness/images/ui-bg_glass_65_ffffff_1x400.png]

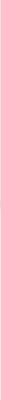

Supplement: Additional file 4 — site_media.zip Graphics required for web GUI interface elements [file 1751-0473-6-9-S4.ZIP › site_media/css/smoothness/images/ui-bg_glass_75_dadada_1x400.png]

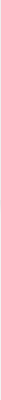

Supplement: Additional file 4 — site_media.zip Graphics required for web GUI interface elements [file 1751-0473-6-9-S4.ZIP › site_media/css/smoothness/images/ui-bg_glass_75_e6e6e6_1x400.png]

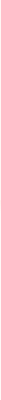

Supplement: Additional file 4 — site_media.zip Graphics required for web GUI interface elements [file 1751-0473-6-9-S4.ZIP › site_media/css/smoothness/images/ui-bg_glass_95_fef1ec_1x400.png]

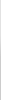

Supplement: Additional file 4 — site_media.zip Graphics required for web GUI interface elements [file 1751-0473-6-9-S4.ZIP › site_media/css/smoothness/images/ui-bg_highlight-soft_75_cccccc_1x100.png]

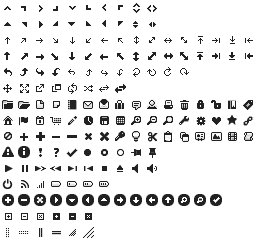

Supplement: Additional file 4 — site_media.zip Graphics required for web GUI interface elements [file 1751-0473-6-9-S4.ZIP › site_media/css/smoothness/images/ui-icons_222222_256x240.png]

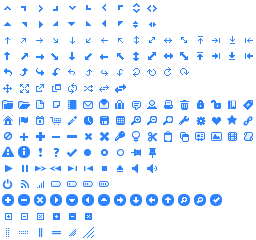

Supplement: Additional file 4 — site_media.zip Graphics required for web GUI interface elements [file 1751-0473-6-9-S4.ZIP › site_media/css/smoothness/images/ui-icons_2e83ff_256x240.png]

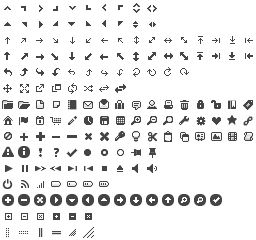

Supplement: Additional file 4 — site_media.zip Graphics required for web GUI interface elements [file 1751-0473-6-9-S4.ZIP › site_media/css/smoothness/images/ui-icons_454545_256x240.png]

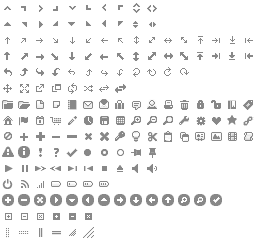

Supplement: Additional file 4 — site_media.zip Graphics required for web GUI interface elements [file 1751-0473-6-9-S4.ZIP › site_media/css/smoothness/images/ui-icons_888888_256x240.png]

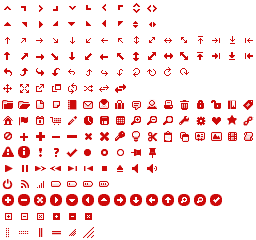

Supplement: Additional file 4 — site_media.zip Graphics required for web GUI interface elements [file 1751-0473-6-9-S4.ZIP › site_media/css/smoothness/images/ui-icons_cd0a0a_256x240.png]

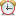

Supplement: Additional file 4 — site_media.zip Graphics required for web GUI interface elements [file 1751-0473-6-9-S4.ZIP › site_media/images/alarm-clock.png]

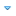

Supplement: Additional file 4 — site_media.zip Graphics required for web GUI interface elements [file 1751-0473-6-9-S4.ZIP › site_media/images/arrow-down.png]

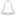

Supplement: Additional file 4 — site_media.zip Graphics required for web GUI interface elements [file 1751-0473-6-9-S4.ZIP › site_media/images/bell-disable.png]

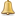

Supplement: Additional file 4 — site_media.zip Graphics required for web GUI interface elements [file 1751-0473-6-9-S4.ZIP › site_media/images/bell.png]

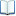

Supplement: Additional file 4 — site_media.zip Graphics required for web GUI interface elements [file 1751-0473-6-9-S4.ZIP › site_media/images/book-open.png]

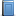

Supplement: Additional file 4 — site_media.zip Graphics required for web GUI interface elements [file 1751-0473-6-9-S4.ZIP › site_media/images/book.png]

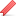

Supplement: Additional file 4 — site_media.zip Graphics required for web GUI interface elements [file 1751-0473-6-9-S4.ZIP › site_media/images/bookmark.png]

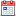

Supplement: Additional file 4 — site_media.zip Graphics required for web GUI interface elements [file 1751-0473-6-9-S4.ZIP › site_media/images/calendar-select.png]

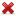

Supplement: Additional file 4 — site_media.zip Graphics required for web GUI interface elements [file 1751-0473-6-9-S4.ZIP › site_media/images/cross.png]

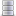

Supplement: Additional file 4 — site_media.zip Graphics required for web GUI interface elements [file 1751-0473-6-9-S4.ZIP › site_media/images/database.png]

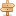

Supplement: Additional file 4 — site_media.zip Graphics required for web GUI interface elements [file 1751-0473-6-9-S4.ZIP › site_media/images/direction.png]

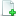

Supplement: Additional file 4 — site_media.zip Graphics required for web GUI interface elements [file 1751-0473-6-9-S4.ZIP › site_media/images/documentplus.png]

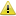

Supplement: Additional file 4 — site_media.zip Graphics required for web GUI interface elements [file 1751-0473-6-9-S4.ZIP › site_media/images/exclamation.png]

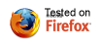

Supplement: Additional file 4 — site_media.zip Graphics required for web GUI interface elements [file 1751-0473-6-9-S4.ZIP › site_media/images/firefox.png]

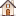

Supplement: Additional file 4 — site_media.zip Graphics required for web GUI interface elements [file 1751-0473-6-9-S4.ZIP › site_media/images/home.png]

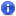

Supplement: Additional file 4 — site_media.zip Graphics required for web GUI interface elements [file 1751-0473-6-9-S4.ZIP › site_media/images/information.png]

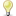

Supplement: Additional file 4 — site_media.zip Graphics required for web GUI interface elements [file 1751-0473-6-9-S4.ZIP › site_media/images/light-bulb.png]

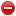

Supplement: Additional file 4 — site_media.zip Graphics required for web GUI interface elements [file 1751-0473-6-9-S4.ZIP › site_media/images/minus-circle.png]

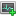

Supplement: Additional file 4 — site_media.zip Graphics required for web GUI interface elements [file 1751-0473-6-9-S4.ZIP › site_media/images/monitor-plus.png]

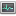

Supplement: Additional file 4 — site_media.zip Graphics required for web GUI interface elements [file 1751-0473-6-9-S4.ZIP › site_media/images/monitor.png]

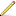

Supplement: Additional file 4 — site_media.zip Graphics required for web GUI interface elements [file 1751-0473-6-9-S4.ZIP › site_media/images/pencil.png]

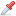

Supplement: Additional file 4 — site_media.zip Graphics required for web GUI interface elements [file 1751-0473-6-9-S4.ZIP › site_media/images/pipette.png]

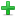

Supplement: Additional file 4 — site_media.zip Graphics required for web GUI interface elements [file 1751-0473-6-9-S4.ZIP › site_media/images/plus.png]

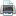

Supplement: Additional file 4 — site_media.zip Graphics required for web GUI interface elements [file 1751-0473-6-9-S4.ZIP › site_media/images/printer.png]

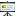

Supplement: Additional file 4 — site_media.zip Graphics required for web GUI interface elements [file 1751-0473-6-9-S4.ZIP › site_media/images/projection-screen-presentation.png]

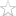

Supplement: Additional file 4 — site_media.zip Graphics required for web GUI interface elements [file 1751-0473-6-9-S4.ZIP › site_media/images/star-empty.png]

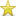

Supplement: Additional file 4 — site_media.zip Graphics required for web GUI interface elements [file 1751-0473-6-9-S4.ZIP › site_media/images/star.png]

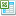

Supplement: Additional file 4 — site_media.zip Graphics required for web GUI interface elements [file 1751-0473-6-9-S4.ZIP › site_media/images/table-excel.png]

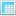

Supplement: Additional file 4 — site_media.zip Graphics required for web GUI interface elements [file 1751-0473-6-9-S4.ZIP › site_media/images/table.png]

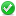

Supplement: Additional file 4 — site_media.zip Graphics required for web GUI interface elements [file 1751-0473-6-9-S4.ZIP › site_media/images/tick-circle.png]

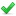

Supplement: Additional file 4 — site_media.zip Graphics required for web GUI interface elements [file 1751-0473-6-9-S4.ZIP › site_media/images/tick.png]

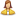

Supplement: Additional file 4 — site_media.zip Graphics required for web GUI interface elements [file 1751-0473-6-9-S4.ZIP › site_media/images/user-orange-female.png]

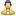

Supplement: Additional file 4 — site_media.zip Graphics required for web GUI interface elements [file 1751-0473-6-9-S4.ZIP › site_media/images/user-orange.png]

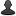

Supplement: Additional file 4 — site_media.zip Graphics required for web GUI interface elements [file 1751-0473-6-9-S4.ZIP › site_media/images/user-silhouette.png]
