# Supplementary material for: LimsPortal and BonsaiLIMS: development of a lab information management system for translational medicine
Source: Source Code Biol Med. 2011 May 13;6:9. doi: 10.1186/1751-0473-6-9 (PMC3113716; doi:10.1186/1751-0473-6-9)

# **BonsaiLIMS**

# A lightweight LIMS system

# Managing Projects

When you enter Bonsai LIMS you will see a listing for the projects. **BonsaiLIMS** is not limited to single projects, you can manage as many projects as you like. An example of a the start screen or project listing is shown below.


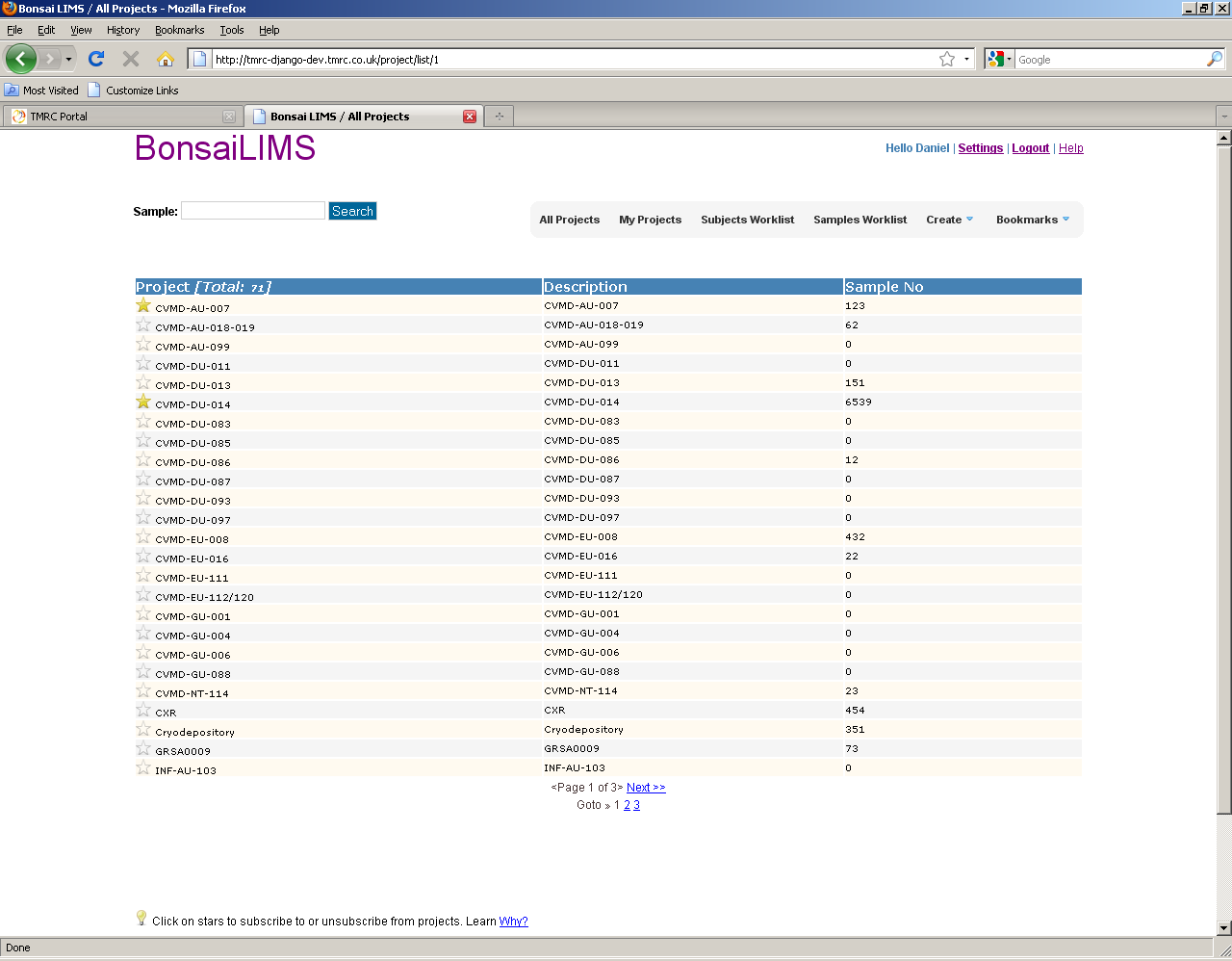


The project name and Description can be set to what ever the user requires. The number of samples associated with the project are shown on the right hand column. Projects can be set up in advance of receiving samples and so will have a sample number of zero in this column. On the very left hand column, next to each project is a star. Users can select these stars by clicking on them, and they will turn Gold. This registers the user for that specific project so they will only see the projects they are registered for in the “My Projects” Tab. This can be helpful is the institution has large numbers of projects.

# Managing subjects

**BonsaiLIMS** makes it easy to manage subjects associated with your projects


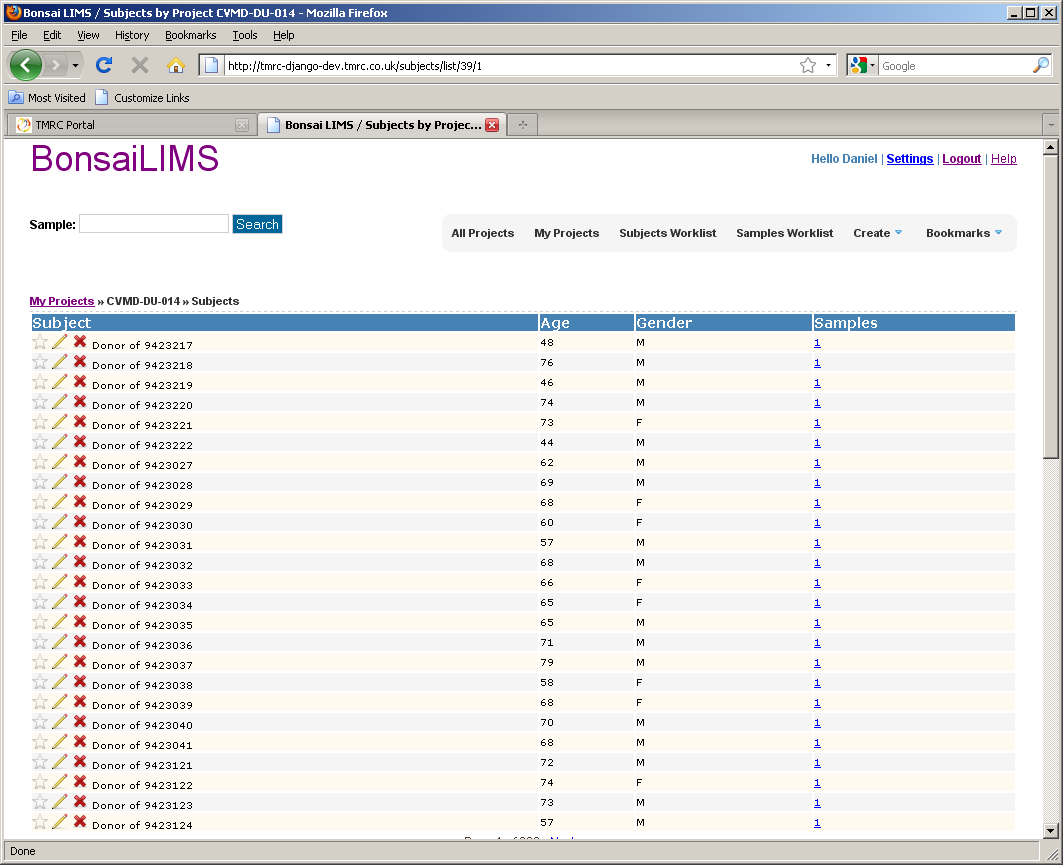


The use of the “create” option allows new subject samples or analysis to be added quickly and easily

#
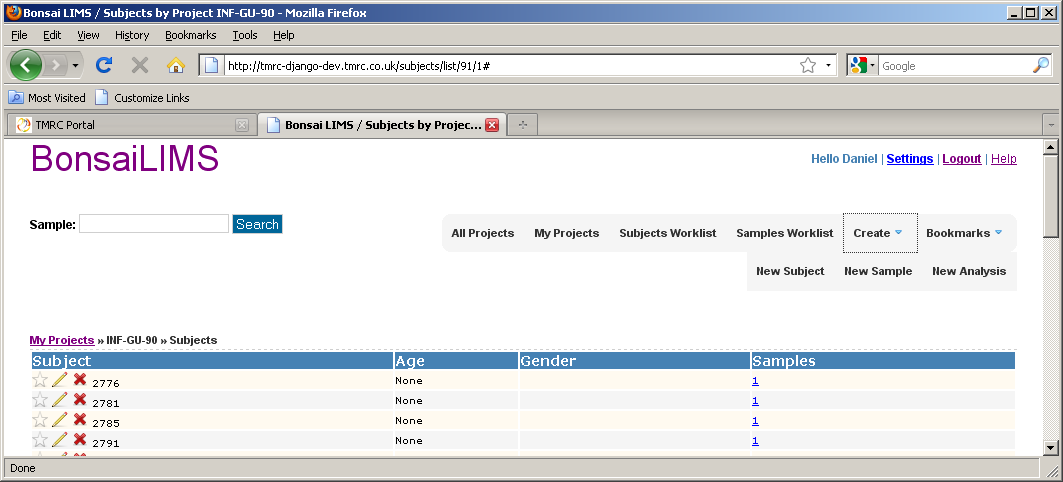


# Managing Samples

**BonsaiLIMS** makes it easy to manage samples associated with your project


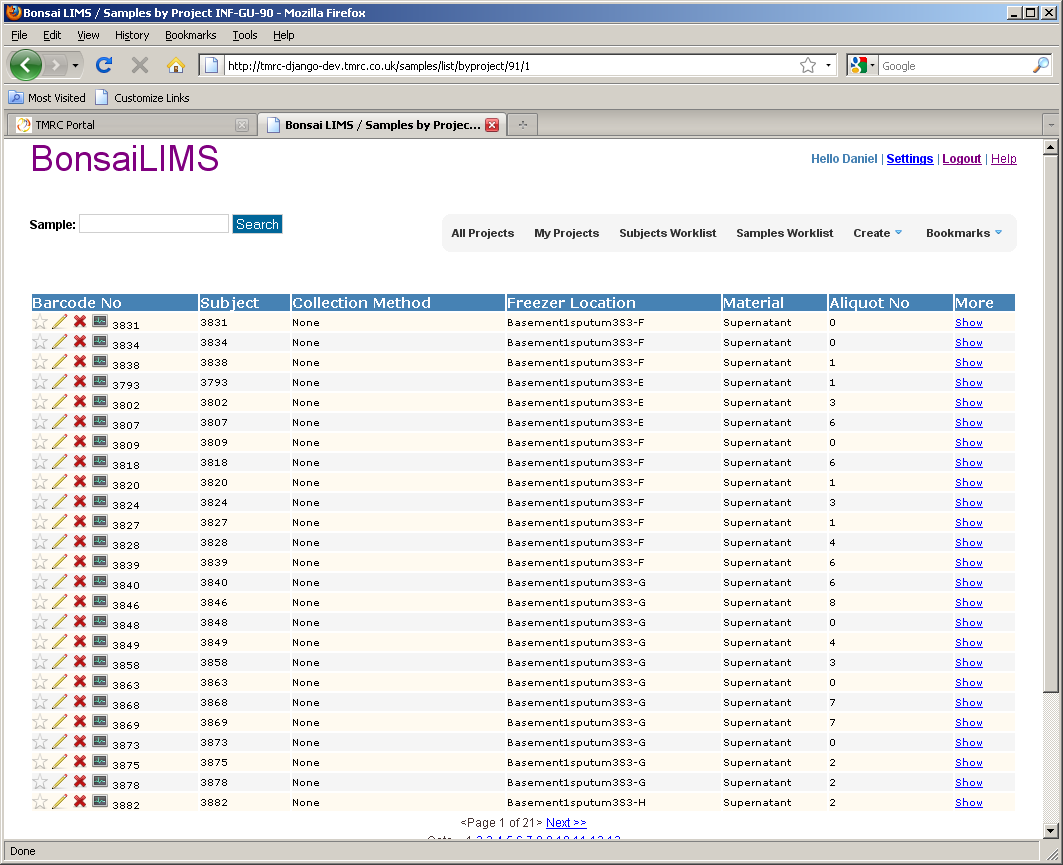


The material types for biomarker work flows associated with transcriptomics or proteomics work flows can be modeled. Individual aliquot numbers can be reported as well as freezer locations and details about sample destruction etc.

# Quickly Finding Things?

**BonsaiLIMS** lets you bookmark and subscribe to projects, subjects and samples. It can be useful for quickly navigating to frequently accessed items. Bookmarks can be set at the level of projects…….


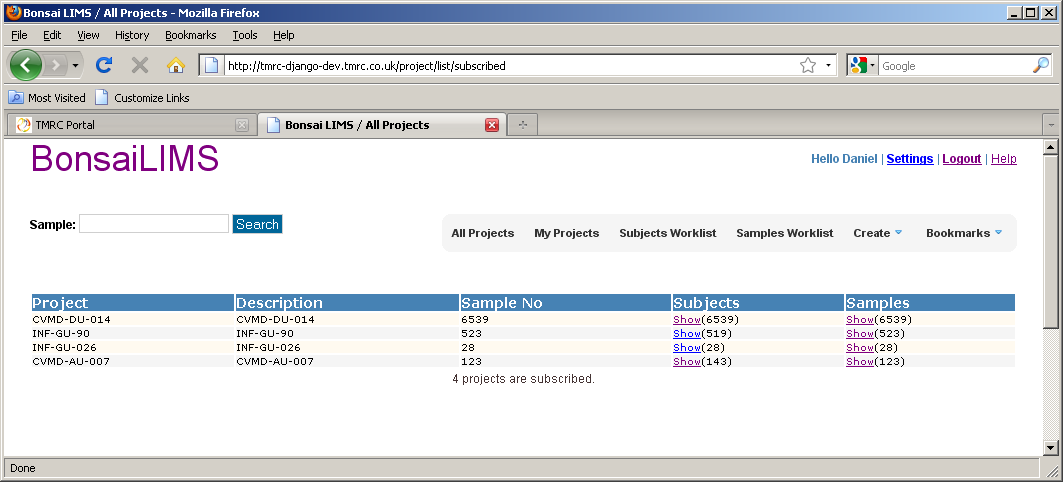


or subjects or samples.


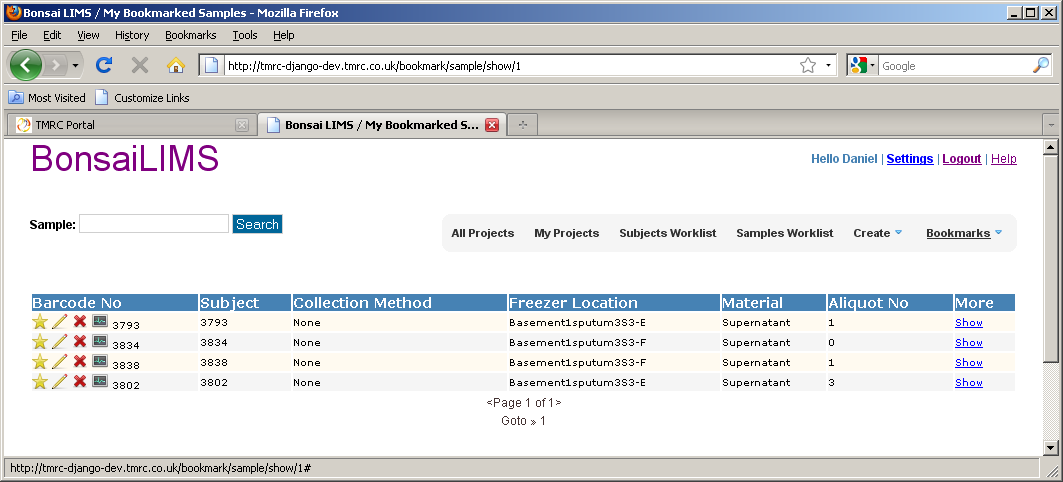

Supplement: Additional file 5 — BonsaiLIMS user guide.doc A brief description of some of the BonsaiLIMS functionality [file 1751-0473-6-9-S5.DOC]
